# Binary Sex Input Has No Effect on Metabolic or Pulmonary Variables: A Within-Subjects Observational Study

**DOI:** 10.3390/sports13080241

**Published:** 2025-07-23

**Authors:** Olivia R. Perez, Michael W. H. Wong, Dustin W. Davis, James W. Navalta

**Affiliations:** Department of Kinesiology and Nutrition Sciences, University of Nevada, Las Vegas, Las Vegas, NV 89154, USA; michael.wong@unlv.edu (M.W.H.W.); dustin.davis@unlv.edu (D.W.D.); james.navalta@unlv.edu (J.W.N.)

**Keywords:** metabolic analysis, gender-diverse inclusion, energy expenditure, sex input

## Abstract

Metabolic analysis systems require binary sex input, conflating biological sex with gender, limiting inclusivity. This study aimed to determine whether sex input altered metabolic or pulmonary variables during self-paced walking and running. Twenty adults completed two 5-min walking and running trials under both female (FC) and male (MC) input conditions in randomized order. Dependent *t*-tests determined differences between conditions; *p*-values < 0.05 were considered significant, and effect sizes were calculated. No significant within-participant differences were found between FC and MC for any variable. During walking, mean relative VO_2_ (mL/kg/min) was 11.13 ± 2.73 (FC) and 10.81 ± 2.39 (MC), *p* = 0.08, *R*^2^ = 0.93; mean energy expenditure (kcal) was 18.28 ± 4.74 (FC) and 17.86 ± 4.33 (MC), *p* = 0.12, *R*^2^ = 0.94. During running, mean relative VO_2_ was 28.80 ± 5.89 (FC) and 28.82 ± 6.06 (MC), *p* = 0.90, *R*^2^ = 0.98; mean energy expenditure was 45.79 ± 13.08 (FC) and 45.55 ± 12.26 (MC), *p* = 0.99, *R*^2^ = 0.98. Binary sex input in the TrueOne 2400 system did not affect variables, supporting inclusive sex and gender data collection to improve research ethics, accuracy, and representation of gender-diverse people without compromising integrity.

## 1. Introduction

Indirect calorimetry is a method used to estimate energy expenditure (EE) without directly measuring heat production [1]. This technique involves measuring gas exchange, specifically the amount of oxygen consumed (VO_2_) and carbon dioxide produced (VCO_2_) during respiration, allowing for the calculation of substrates oxidized for energy (i.e., carbohydrates, fats, proteins). This information is then used to indirectly estimate EE.

Scientific studies in kinesiology routinely involve indirect calorimetry. A popular device is the ParvoMedics TrueOne 2400 (Sandy, UT, USA), a computerized metabolic analysis system that is a non-breath-by-breath system that utilizes a mixing chamber to analyze gas exchange [2]. The device is widely regarded as the gold-standard metabolic analysis system for measuring inspiratory and expiratory gas exchange variables in modern sport and exercise science laboratories, with several studies supporting its validity and reliability [2,3,4]. Given its reputation, the TrueOne 2400 is consistently used in comparative studies to analyze new and developing metabolic analysis systems and methods [5,6].

Though recognized as valid and reliable, the TrueOne 2400 requires users to input participant sex within a binary framework—specifically selecting either “female” or “male”—a constraint referred to here as binary sex input. Sex encompasses biological factors (e.g., genetic, physiological, and anatomical characteristics), whereas gender is a social construct encompassing identity, roles, and behaviors acquired through socialization [7]. The reliance on binary sex input is not unique to the TrueOne 2400; many metabolic analysis systems similarly restrict users to binary categories or conflate sex and gender by mislabeling sex as gender and offering options such as “female” or “male” rather than gendered terms like “woman” or “man.” The COSMED K5 Omnia software (Rome, Italy) utilized in a recent study required that participants enter gender but only offered the binary sex options “female” and “male” via a dropdown menu [8]. This software design convention restricts inclusivity and usability for people whose sex or gender identity does not align with the binary categories, particularly in research and clinical applications.

Despite the TrueOne 2400’s prominence in kinesiology, it remains unknown whether the selection of binary sex input meaningfully alters measurements of metabolic or pulmonary variables. The structure of the device’s software suggests a theoretical assumption that user-provided sex data influence internal calculations or output variables. To date, no published study has tested this assumption in the TrueOne 2400. A recent within-subjects analysis using the COSMED K5, another leading metabolic system in the field, found no effect of sex input on measured variables and recommended that similar studies be conducted across devices [8]. Addressing this gap, the objective of the present study was to determine whether binary sex input affects measured metabolic or pulmonary variables during self-paced walking and running using the ParvoMedics TrueOne 2400. We hypothesized that sex input would have no significant effect on measured variables.

## 2. Materials and Methods

### 2.1. Participants

Effect sizes from our work on the COSMED K5 (lowest *R*^2^ = 0.54) using the same study design were used to determine the number of participants needed in the current investigation [8]. An a priori power analysis was completed in G*Power 3.1.9.7 (Düsseldorf, North Rhine-Westphalia, Germany) [9] utilizing linear multiple regression: fixed model, *R*^2^ deviation from zero, an α error of probability of 0.05, and a power (1 − β error of probability) of 0.95, indicating that a total sample size of twenty participants was needed for 95% statistical power. The Institutional Review Board (#2023-525) approved the investigation, which was conducted in full compliance with the *International Journal of Exercise Science’s* ethical criteria. 

Twenty adult participants (self-identified sex: female *n* = 10, male *n* = 10, identified otherwise *n* = 0) participated in this study after completing an online informed consent form via Qualtrics (Provo, UT, USA), along with a health risk questionnaire to determine eligibility. Participants were recruited from the University of Nevada, Las Vegas campus and surrounding communities. Participants’ demographic information (arithmetic mean ± standard deviation) included age (years) 24.5 ± 7.5, height (centimeters) 168.3 ± 9.2, and body mass (kilograms) 68.8 ± 13.7.

### 2.2. Protocol

A randomization chart (Google Sheets, Mountain View, CA, USA) was developed and utilized to determine the order in which participants were assigned the binary female or male sex (called the female condition and male condition, respectively) in the participant profile of the ParvoMedics TrueOne 2400 metabolic analysis system (Sandy, UT, USA). A Polar H10 heart rate monitor (Polar Electro Inc., Kempele, Finland) was utilized in conjunction with the TrueOne 2400 to collect heart rate data. Metabolic and pulmonary variables included relative VO_2_ (mL·kg^−1^·min^−1^), absolute VO_2_ (L·min^−1^), VCO_2_ (L·min^−1^), ventilation (VE [L·min^−1^]), respiratory exchange ratio (RER), respiratory rate in breaths per minute (RR [BPM]), and EE (kcal).

Each participant completed the study on a single testing day. Resting heart rate was recorded by having participants sit quietly in a comfortable position for a minimum of five minutes, during which participants’ heart rate was continually monitored until it stabilized with no interruptions. The resting heart rate was determined utilizing the lowest observed heart rate during this period. Then, participants were fitted for a facemask, chosen for comfort and because no differences in metabolic data were seen when compared to a traditional mouthpiece [10].

Before securing the facemask for testing, participants determined their preferred treadmill speeds (WOODWAY 4Front, Waukesha, WI, USA) for walking and running using a blinded approach consisting of three self-paced trials per gait [11]. During each trial, participants were blinded to the displayed treadmill speed and instructed to gradually increase it from 0 m·min^−1^ to a pace they deemed comfortable and sustainable for walking or running, then notify the researchers. Researchers manually recorded the speed at that point, after which participants gradually decreased the speed back to 0 m·min^−1^. This procedure was completed three times for walking and three times for running. For each participant, the recorded speeds from the three trials per gait were averaged separately to determine individualized walking and running speeds. Each participant used their individualized walking and running speeds for all subsequent testing. The sample mean speeds were 64.9 ± 18.2 m·min^−1^ for walking and 130.2 ± 29.0 m·min^−1^ for running.

**Figure 1:**
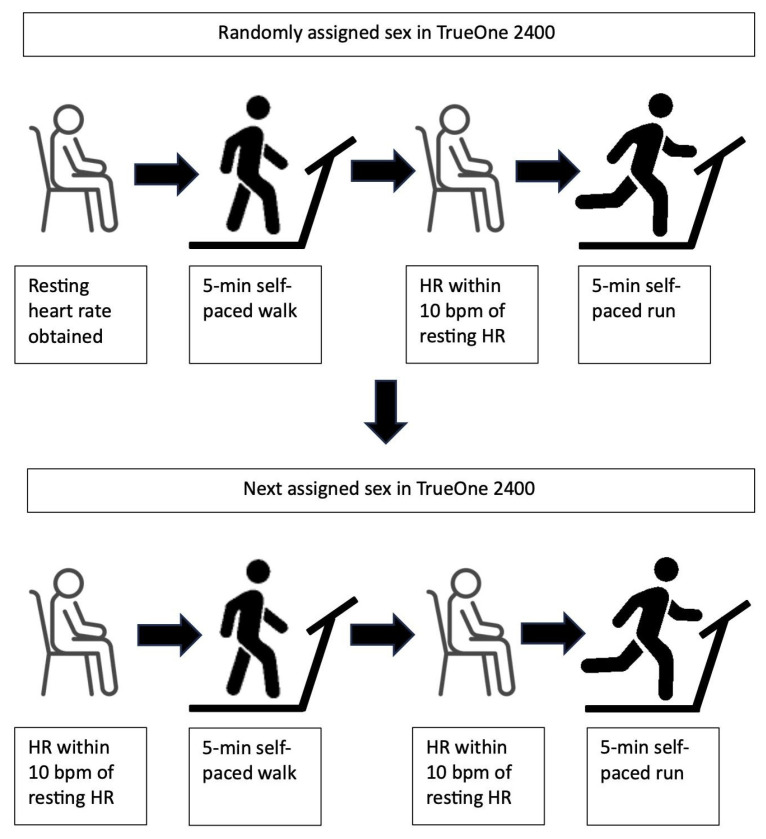
Study protocol illustrating the sequence of participant activity under two software-assigned sex conditions in the ParvoMedics TrueOne 2400 metabolic analysis system. In the first condition (randomized to either “female” or “male”), participants sat to establish resting heart rate, completed a 5-min self-paced walk at their individualized preferred speed, rested until heart rate returned within 10 beats per minute of resting, and completed a 5-min self-paced run. After a seated recovery until heart rate was again within 10 beats per minute of resting, participants completed the same walk-rest-run sequence under the other sex condition. HR = heart rate. Artist: Nigar Novruxova (vecteezy.com). Accessed on 11 November 2024.

The facemask was secured on the participant, and after confirming no air leakage, the TrueOne 2400 tubing was firmly attached. Each participant first walked for five minutes and then ran for five minutes under the female and male conditions (see Figure 1). Between walking and running, participants were guided off the treadmill and sat until their heart rate was within 10 beats per minute of their resting heart rate (mean duration = 3.6 ± 3.9 min). When the first condition was completed, a new participant profile was created for the next sex utilizing the same age, height, and body mass. The protocol was repeated after participants sat until their heart rate was within 10 beats per minute of their resting rate (mean duration = 6.3 ± 3.5 min).

### 2.3. Statistical Analysis

During the preparation of this manuscript/study, the author(s) used ChatGPT 4o, 4.5, and o3 for the purposes of analyzing the data and refining the clarity of the writing, specifically in the abstract, introduction, methods, and discussion. The authors have reviewed and edited the output to verify its accuracy and take full responsibility for the content of this publication.

Data were checked for normality using the Shapiro-Wilk test (IBM SPSS Statistics, Version 29.0.2.0, Armonk, NY, USA). Dependent *t*-tests for each dependent variable were conducted to determine if there were differences in metabolic and pulmonary variables between conditions. Comparisons that violated the assumption of normality were evaluated using the Wilcoxon Signed Rank Test instead. *p*-values < 0.05 were considered significant. Effect sizes for *t*-tests were calculated as Cohen’s *d*, with negligible = 0.0–0.2, small = 0.2–0.49, medium = 0.5–0.79, and large ≥ 0.8; or the Wilcoxon Signed Rank Test through *r*, with negligible = 0.0–0.09, small = 0.1–0.29, medium = 0.3–0.49, and large ≥ 0.5 [12]. Correlations between data from the female and male conditions were evaluated using Pearson product-moment correlation coefficients (*r*) for each dependent variable and the coefficient of determination (*R*^2^) as the effect size. Thresholds for correlation coefficients were interpreted as perfect = 1.0, very strong = 0.8–0.9, moderate = 0.6–0.79, fair = 0.3–0.59, poor = 0.1–0.29, and none = 0 [13].

## 3. Results

No within-participant differences between the female and male conditions were observed for any metabolic or pulmonary variables during self-paced walking (see Table 1). The shared variance was between 36% and 95% (see Table 1).

Similar to walking, no within-participant differences between the female and male conditions were observed for any metabolic or pulmonary variables during self-paced running (see Table 2). The shared variance was between 61% and 98% (see Table 2).

Based on the randomization scheme, 14 participants (*n* = 7 female, *n* = 7 male) began the study under the condition aligning with their self-identified sex (i.e., female participant first randomized into the female condition), while 6 participants (*n* = 3 female, *n* = 3 male) began the study under the condition not aligning with their self-identified sex (i.e., female participant first randomized into the male condition). No within-participant differences for any variable were observed when the sex-aligned condition was compared to the sex-not-aligned condition for self-paced walking (see Table 3) or running (see Table 4).

The present study did not aim to evaluate differences in metabolic and pulmonary variables between female and male participants (sex differences), so these data were not analyzed. However, to align with the Sex and Gender Equity in Research (SAGER) guidelines [14] and support future meta-analyses, we present disaggregated metabolic and pulmonary data when participants’ self-identified sex aligned with the sex condition, and when it did not align (see Table 5).

## 4. Discussion

The present study aimed to determine whether inputting “female” or “male” as the participants’ sex in the ParvoMedics TrueOne 2400 metabolic analysis system influenced metabolic or pulmonary variables during self-paced walking and running. Based on existing evidence indicating validity and reliability of the TrueOne 2400 for measuring respiratory gases and energy expenditure [2,3,4], we hypothesized that no meaningful differences would arise from altering sex input within the binary framework (female-male). Consistent with our expectations, no statistically significant within-participant differences emerged across a range of metabolic (VO_2_, VCO_2_, EE) and pulmonary variables (VE, RER, RR) during either walking or running. The robustness of this finding is supported by the high correlations observed between sex conditions (*r* > 0.90 for all variables except RER and RR), indicating negligible effects of sex input on the measured variables within the bounds of this study’s sample size and design. We believe deviation between conditions to be due to the normal physiological variation that occurs between bouts of exercise [15,16]. Our findings provide novel evidence, to our knowledge the first of its kind, indicating that the TrueOne 2400 software’s built-in requirement of inputting sex as either female or male offers no discernible physiological or analytical benefit. More importantly, mandating binary sex input may constitute an unnecessary restriction, potentially acting as a subtle but meaningful barrier to participation for people whose sex or gender identities fall outside these categories. Consequently, this requirement could unintentionally contribute to exclusion within sport and exercise science research and practice.

While we showed that binary sex input did not influence within-participant measurements of metabolic or pulmonary variables, literature has reported between-participant differences between presumably cisgender females and males during exercise. Sex differences in endurance exercise performance have been explained by a difference in VO_2_max [17]. The inability of elite female athletes to match oxygen consumption of elite male athletes is attributed to central factors, as females generally have smaller hearts, lungs, and lower hemoglobin mass than males, which limits the ability to deliver oxygen to working musculature [18]. When submaximal exercise was considered (30%, 50%, 70%, and 90% VO_2_max), sex differences were not observed in participants who were matched for VO_2_max and training background [19]. When considering pulmonary physiology during exercise, it has been argued that three morphological differences account for sex differences: luminal area of the central conducting airways, lung size, and the shape of the ribcage and lungs [20]. Thibault et al. have argued that a 10.7% physiological difference exists between the highest performing females and males in various events [21]. An evaluation of running velocities among top finishers of the New York City marathon reported that the sex gap widened with increasing age, a phenomenon likely influenced by lower female participation rates [22]. Conversely, we demonstrated that the sex gap in running velocities narrowed significantly when female representation was equal to or exceeded that of males in a trail running environment [23]. Contrasting findings with sex differences between females and males in sport and exercise research might be inflated or obscured by unequal representation and imbalanced group sizes. Such systemic underrepresentation of female participants contributes to persistent knowledge gaps, potentially distorting interpretations of sex differences [24,25,26]. Moreover, an even greater gap exists for populations outside the female-male binary, with negligible data currently available for intersex, transgender, non-binary, or gender-diverse people [24,27,28]. This lack of inclusive data collection methods limits the accuracy and applicability of exercise recommendations for gender-expansive people and risks significant misinterpretation due to misgendering participants [27]. Given the context of our findings, we suggest that because pulmonary and metabolic data do not seem to be influenced by sex input, these measures represent absolute values returned for people of all genders. Unless an investigation is designed to specifically test for gender differences, the output of these variables can be considered a true representation (i.e., not sex- or gender- dependent) and studies inclusive of all individuals can be conducted.

While the present study provides insight into how sex input does not influence metabolic and pulmonary variables returned from the TrueOne 2400, several limitations should be considered. First, no participants identified outside of the binary framework, which may suggest our methods of recruitment and enrollment deterred participation by gender-diverse people or were insufficient to reach them. We acknowledge this limitation and emphasize the need for future research to adopt more inclusive recruiting strategies and community engagement practices that actively represent the gap of gender-diverse populations in sport and exercise science research. Second, as participants self-selected their preferred walking and running speeds, there was between-participant variability in chosen intensities and rest periods needed between trials for heart rate to return to resting. However, such variability did not affect our statistical analyses, which compared participants to themselves. Third, the exercise trials in this study were conducted in a controlled laboratory setting, which limits the ability to generalize our findings to real-world settings. Finally, findings are device-specific until replicated with other metabolic analysis systems or direct calorimetry. Despite the acknowledged limitations, the present study has notable strengths that support its scientific rigor and relevance. The sample size (*N* = 20) aligns closely with established standards for validity studies in exercise science, including recent wearable technology validation protocols [29]. Additionally, employing a within-participant design, wherein each participant completed identical self-paced walking and self-paced running conditions under both female and male settings, markedly strengthened internal validity and allowed robust direct comparisons. Also noteworthy is our adherence to the SAGER guidelines [14] by clearly disaggregating data according to participants’ self-identified sex, thus contributing to greater transparency and inclusivity in reporting and facilitating future meta-analyses.

To advance the generalizability and inclusivity of metabolic research, future studies should replicate and extend these findings across varied exercise modalities, intensities, populations, and metabolic analysis systems. Researchers are encouraged to reconsider restrictive binary sex input requirements and adopt inclusive, participant-centered approaches to collecting sex and gender data, such as self-report fields with open-ended or expanded response options. These practices increase the precision of recorded participant characteristics, reduce exclusion by allowing people to represent themselves authentically, and may help increase participation among historically marginalized groups. Addressing the lack of data on intersex, transgender, non-binary, and gender-diverse individuals in sport and exercise science is essential to building a more representative and equitable evidence base [27].

We acknowledge, however, that integrating expanded sex and gender responses into device calibration and statistical analysis presents practical challenges. In the context of device calibration, developers could use relevant anatomical parameters (e.g., lung volume, fat-free mass, body surface area) rather than relying on sex as a proxy. For statistical analysis, researchers may code open-ended responses post hoc using established frameworks that align with best practices in sex and gender research, enabling appropriate stratification or covariate adjustment. For example, the National Institutes of Health has the robust *All of Us* dataset [30], that includes data from Fitbit devices, on participants who self-identified across a range of sexes and genders (cisgender female, cisgender male, transgender, non-binary, additional options). Importantly, inclusive data collection does not have to compromise analytical rigor; rather, it demands thoughtful design and transparency in how identity-related variables are operationalized [14].

Consistent with the SAGER guidelines [14], sex and gender should be accurately reported and meaningfully integrated into study design, data interpretation, and dissemination. These methodological improvements must be paired with robust ethical safeguards to protect the confidentiality of sensitive identity-related information. As metabolic technologies and wearable systems become more widely adopted in both research and applied settings, inclusive data practices are not simply preferable—they are necessary for advancing scientifically valid and socially responsible research.

## 5. Conclusions

In summary, the present within-subjects study found that binary sex input (“female” or “male”) in the ParvoMedics TrueOne 2400 metabolic analysis system did not alter measured metabolic or pulmonary variables during self-paced walking and running. These findings indicate that sex input, as currently required by the device software, has no measurable effect on physiological outputs under the conditions tested.

## Figures and Tables

**Table 1:** Metabolic and pulmonary variables during self-paced walking when sex was input as female and when sex was input as male.

	Female Condition	Male Condition	*p*-Value	Cohen’s *d*	Pearson’s *r*	*R* ^2^
VO_2_ (mL·kg^−1^·min^−1^)	11.13 ± 2.73 (9.8, 12.4)	10.81 ± 2.39 (9.7, 11.9)	0.08	0.408	0.962	0.9254
VO_2_ (L·min^−1^)	0.76 ± 0.20 (0.66, 0.85)	0.73 ± 0.18 (0.65, 0.82)	0.05	0.479	0.975	0.9506
VCO_2_ (L·min^−1^)	0.62 ± 0.17 (0.54, 0.7)	0.60 ± 0.16 (0.53, 0.68)	0.07	0.436	0.969	0.9390
VE (L·min^−1^)	20.36 ± 5.35 (17.9, 22.9)	19.28 ± 4.62 (17.1, 21.4)	0.89	0.032 (*r*)	0.908	0.8245
RER	0.82 ± 0.08 (0.79, 0.86)	0.82 ± 0.07 (0.79, 0.85)	0.74	0.074	0.603	0.3636
RR (bpm)	20.07 ± 5.58 (17.8, 22.4)	19.74 ± 5.58 (17.1, 22.3)	0.70	0.088	0.750	0.5625
EE (kcal)	18.28 ± 4.74 (16.1, 20.5)	17.86 ± 4.33 (15.8, 19.9)	0.12	0.361	0.970	0.9409

VO_2_: oxygen consumed; VCO_2_: carbon dioxide produced; VE: ventilation; RER: respiratory exchange ratio; RR: respiratory rate in breaths per minute (bpm); EE: energy expenditure; kcal: kilocalories. (*r*) in the Cohen’s *d* column is the effect size for the Wilcoxon Signed Rank Test. Cohen’s *d* interpreted as negligible (0.0–0.2), small (0.2–0.49), medium (0.5–0.79), and large (≥0.8). For VE’s effect size, *r* interpreted as negligible (0.0–0.09), small (0.1–0.29), medium (0.3–0.49), and large (≥0.5) [12]. Correlation coefficients interpreted as perfect (1.0), very strong (0.8–0.9), moderate (0.6–0.79), fair (0.3–0.59), poor (0.1–0.29), and none (0) [13].

**Table 2:** Metabolic and pulmonary variables during self-paced running when sex was input as female and when sex was input as male.

	Female Condition	Male Condition	*p*-Value	Cohen’s *d*	Pearson’s *r*	*R* ^2^
VO_2_ (mL·kg^−1^·min^−1^)	28.80 ± 5.89 (26.0, 31.6)	28.82 ± 6.06 (26.0, 31.7)	0.90	0.03	0.990	0.980
VO_2_ (L·min^−1^)	1.96 ± 0.52 (1.72, 2.2)	1.96 ± 0.49 (1.73, 2.19)	0.84	0.05 (*r*)	0.993	0.986
VCO_2_ (L·min^−1^)	1.82 ± 0.58 (1.55, 2.09)	1.84 ± 0.56 (1.57, 2.1)	0.56	0.13 (*r*)	0.984	0.968
VE (L·min^−1^)	52.10 ± 19.40 (43.0, 61.2)	53.26 ± 15.94 (45.8, 60.7)	0.05	0.44 (*r*)	0.970	0.941
RER	0.92 ± 0.08 (0.88, 0.96)	0.93 ± 0.08 (0.89, 0.97)	0.51	0.15 (*r*)	0.782	0.612
RR (bpm)	31.54 ± 9.56 (27.1, 36.0)	32.22 ± 8.46 (28.3, 36.2)	0.53	0.14	0.870	0.757
EE (kcal)	45.79 ± 13.08 (39.7, 51.9)	45.55 ± 12.26 (39.8, 51.3)	0.99	0.004 (*r*)	0.990	0.980

VO_2_: oxygen consumed; VCO_2_: carbon dioxide produced; VE: ventilation; RER: respiratory exchange ratio; RR: respiratory rate in breaths per minute (bpm); EE: energy expenditure; kcal: kilocalories. (*r*) in the Cohen’s *d* column is the effect size for the Wilcoxon Signed Rank Test. Cohen’s *d* interpreted as negligible (0.0–0.2), small (0.2–0.49), medium (0.5–0.79), and large (≥0.8). For VE’s effect size, *r* interpreted as negligible (0.0–0.09), small (0.1–0.29), medium (0.3–0.49), and large (≥0.5) [12]. Correlation coefficients interpreted as perfect (1.0), very strong (0.8–0.9), moderate (0.6–0.79), fair (0.3–0.59), poor (0.1–0.29), and none (0) [13].

**Table 3:** Metabolic and pulmonary variables during self-paced walking when condition aligned with sex and when condition did not align.

	Sex Aligned	Sex Not Aligned	*p*-Value	Cohen’s *d*	Pearson’s *r*	*R* ^2^
VO_2_ (mL·kg^−1^·min^−1^)	11.09 ± 2.60 (9.9, 12.3)	10.85 ± 2.53 (9.7, 12.0)	0.20	0.30	0.951	0.904
VO_2_ (L·min^−1^)	0.75 ± 0.18 (0.67, 0.83)	0.74 ± 0.20 (0.65, 083)	0.34	0.22	0.973	0.947
VCO_2_ (L·min^−1^)	0.61 ± 0.16 (0.54, 0.68)	0.62 ± 0.18 (0.53, 0.70)	0.67	0.10	0.968	0.937
VE (L·min^−1^)	19.53 ± 4.25 (17.5, 21.5)	20.11 ± 5.69 (17.4, 22.8)	0.31	0.24	0.920	0.846
RER	0.81 ± 0.07 (0.78, 0.84)	0.83 ± 0.08 (0.80, 0.87)	0.12	0.36	0.642	0.412
RR (bpm)	19.56 ± 4.38 (17.8, 21.9)	19.95 ± 6.02 (17.1, 22.8)	0.91	0.02	0.780	0.608
EE (kcal)	18.13 ± 4.17 (16.2, 20.1)	18.01 ± 4.88 (15.7, 20.3)	0.69	0.09	0.974	0.949

VO_2_: oxygen consumed; VCO_2_: carbon dioxide produced; VE: ventilation; RER: respiratory exchange ratio; RR: respiratory rate in breaths per minute (bpm); EE: energy expenditure; kcal: kilocalories. Cohen’s *d* interpreted as negligible (0.0–0.2), small (0.2–0.49), medium (0.5–0.79), and large (≥0.8) [12]. Correlation coefficients interpreted as perfect (1.0), very strong (0.8–0.9), moderate (0.6–0.79), fair (0.3–0.59), poor (0.1–0.29), and none (0) [13].

**Table 4:** Metabolic and pulmonary variables during self-paced running when condition aligned with sex and when condition did not align.

	Sex Aligned	Sex Not Aligned	*p*-Value	Cohen’s *d*	Pearson’s *r*	*R* ^2^
VO_2_ (mL·kg^−1^·min^−1^)	28.75 ± 5.83 (26.0, 31.5)	28.87 ± 6.11 (26.0, 31.7)	0.55	0.14	0.991	0.982
VO_2_ (L·min^−1^)	1.95 ± 0.49 (1.73, 2.18)	1.97 ± 0.52 (1.72, 2.21)	0.73	0.08 (*r*)	0.995	0.990
VCO_2_ (L·min^−1^)	1.84 ± 0.56 (1.58, 2.1)	1.82 ± 0.58 (1.54, 2.09)	0.54	0.14 (*r*)	0.984	0.968
VE (L·min^−1^)	52.15 ± 16.41 (44.5, 59.8)	53.22 ± 19.01 (44.3, 62.1)	1.00	0.00 (*r*)	0.961	0.924
RER	0.93 ± 0.08 (0.90, 0.97)	0.92 ± 0.08 (0.88, 0.96)	0.18	0.30 (*r*)	0.793	0.629
RR (bpm)	31.09 ± 8.87 (26.9, 35.2)	32.67 ± 9.13 (28.4, 36.9)	0.13	0.31	0.877	0.769
EE (kcal)	45.48 ± 10.85 (39.8, 51.2)	45.86 ± 12.13 (39.7, 52.0)	0.63	0.11 (*r*)	0.991	0.982

VO_2_: oxygen consumed; VCO_2_: carbon dioxide produced; VE: ventilation; RER: respiratory exchange ratio; RR: respiratory rate in breaths per minute (bpm); EE: energy expenditure; kcal: kilocalories. Cohen’s *d* interpreted as negligible (0.0–0.2), small (0.2–0.49), medium (0.5–0.79), and large (≥0.8). For VE’s effect size, *r* interpreted as negligible (0.0–0.09), small (0.1–0.29), medium (0.3–0.49), and large (≥0.5) [12]. Correlation coefficients interpreted as perfect (1.0), very strong (0.8–0.9), moderate (0.6–0.79), fair (0.3–0.59), poor (0.1–0.29), and none (0) [13].

**Table 5:** Disaggregated metabolic and pulmonary data walking and running in the condition where sex was aligned, and in the condition where sex was not aligned.

	Walking—Sex Aligned		Running—Sex Aligned	
	Female (*n* = 10)	Male (*n* = 10)	Female (*n* = 10)	Male (*n* = 10)
VO_2_ (mL·kg^−1^·min^−1^)	11.00 ± 3.41	11.17 ± 1.63	26.61 ± 5.63	30.89 ± 5.47
VO_2_ (L·min^−1^)	0.68 ± 0.16	0.83 ± 0.16	1.64 ± 0.27	2.27 ± 0.46
VCO_2_ (L·min^−1^)	0.54 ± 0.14	0.68 ± 0.15	1.47 ± 0.24	2.20 ± 0.55
VE (L·min^−1^)	17.94 ± 6.32	21.12 ± 4.41	41.90 ± 7.12	62.40 ± 16.85
RER	0.80 ± 0.08	0.82 ± 0.07	0.90 ± 0.05	0.97 ± 0.08
RR (bpm)	20.74 ± 4.83	18.98 ± 3.95	30.01 ± 10.29	32.18 ± 7.58
EE (kcal)	16.12 ± 3.82	20.24 ± 3.49	37.57 ± 6.48	53.39 ± 11.50
	**Walking—Sex Not Aligned**		**Running—Sex Not Aligned**	
	**Female (*n* = 10)**	**Male (*n* = 10)**	**Female (*n* = 10)**	**Male (*n* = 10)**
VO_2_ (mL·kg^−1^·min^−1^)	10.45 ± 3.02	11.25 ± 2.01	26.57 ± 6.17	30.98 ± 5.56
VO_2_ (L·min^−1^)	0.64 ± 0.15	0.84 ± 0.2	1.65 ± 0.31	2.28 ± 0.51
VCO_2_ (L·min^−1^)	0.53 ± 0.15	0.70 ± 0.17	1.47 ± 0.27	2.16 ± 0.62
VE (L·min^−1^)	17.45 ± 4.26	22.77 ± 5.86	44.13 ± 8.19	62.31 ± 22.64
RER	0.82 ± 0.08	0.85 ± 0.07	0.89 ± 0.04	0.94 ± 0.1
RR (bpm)	20.50 ± 6.98	19.40 ± 5.20	32.26 ± 9.68	33.07 ± 9.05
EE (kcal)	15.48 ± 3.84	20.55 ± 4.62	37.70 ± 6.95	54.01 ± 12.99

VO_2_: oxygen consumed; VCO_2_: carbon dioxide produced; VE: ventilation; RER: respiratory exchange ratio; RR: respiratory rate in breaths per minute (bpm); EE: energy expenditure; kcal: kilocalories.

## Data Availability

The data presented in this study are available on request from the corresponding author.

## Abbreviations

The following abbreviations are used in this manuscript:

BPM	Breaths per minute
Kcal	Kilocalories
RER	Respiration exchange ratio
RR	Respiratory rate
SAGER	Sex and Gender Equity in Research
VCO_2_	Carbon dioxide production
VE	Ventilation
VO_2_	Oxygen consumption
